# Reflections on the need for a vaccine strategy against COVID-19 for pregnant and postpartum women

**DOI:** 10.6061/clinics/2021/e3471

**Published:** 2021-10-28

**Authors:** Fábio Roberto Cabar, Rossana Pulcineli Vieira Francisco

**Affiliations:** Departamento de Obstetricia e Ginecologia, Faculdade de Medicina FMUSP, Universidade de Sao Paulo, Sao Paulo, SP, BR.

The COVID-19 pandemic posed a major unprecedented challenge to national health systems and governments. The risk of infection and its consequences have changed the daily lives of billions of people, with an incalculable economic cost. The pandemic also led to an increased demand for medical and health care; therefore, it was necessary to create guidelines and protocols to indicate treatment priorities and which patients should be treated.

Based on this issue, ethical and bioethical principles were raised and discussed. The “welfare state” is a concept that covers the social, political, and economic areas and sees the state as an institution that is obligated to organize the economy and guarantee basic service access, such as health, education, and security to its citizens, thereby reducing social inequalities. The full functioning of a welfare state demands effective public policies, and the government must ensure that the rights of the population are maintained. In Brazil, one of these policies is the Unified Health System, one of the few health systems in the world that is completely free, regardless of nationality, socioeconomic status, household, or any other factor. This is a public policy in line with the idea of social welfare, as it uses public resources to provide health care to all residents in Brazil. The great difficulty in implementing and operating this system is the need for financial resources.

Some theories hold that there is a moral reason for doing what is good for everyone. This can be called the principle of beneficence. Utilitarians, for example, argue that maximizing what is good for everyone is the ultimate expression of morality. However, some theories defend other principles that should be pursued and valued, without ignoring the issue of the difference between what is fair distribution and what is fair for all citizens of society; therefore, the principle of beneficence must be balanced with the principle of justice (1).

The principle of autonomy values the people’s freedom to choose and determine how to live their lives by themselves. It turns out that individual freedoms may conflict with the general good; this occurs, for example, when citizens disrespect social distancing or when they demand a resource that is scarce and insufficient for everyone for themselves and their families. At this point, it is important to reflect on whether the principle of beneficence should be impartial, providing resources for the good of all, or whether it should be allowed to provide more to those who are close to us. In the context of the pandemic, is it reasonable to assume impartiality?

During a pandemic, a situation in which health systems are experiencing massive challenges, and there is an indisputable need to prioritize the necessity of some citizens, it is not necessary to talk about egalitarianism, as it is impossible to treat all citizens equally. Moreover, it is a mistake not to consider that some actions can generate serious consequences, such as the avoidable loss of many lives, or the ethics of the best way to do the general good.

Several practical rules can guide quick decision-making in such a situation. A rule of utilitarianism is to save as many people as possible, using a screening mechanism: (i) treatment duration; (ii) life expectancy and, indirectly, the patient’s age; (iii) quality of life, i.e., not only how long the patient will live, but how well they will live; (iv) social well-being, so that it is relevant to consider not only the individual benefit for the patient directly benefiting from a conduct, but also the benefit that the person receiving the treatment may offer to other individuals; and, especially, (v) the available financial resources *versus* the cost of each treated patient, as the more resources that are spent on a single treatment, the fewer resources will be available for others (1).

Over the decades, history has taught us lessons about vaccine-preventable diseases in pregnancy. The recognition of the specific conditions of pregnancy and puerperium and the unique consequences of diseases during pregnancy has enabled the development of safe vaccines capable of greatly improving maternal and perinatal prognoses. The use of vaccines during pregnancy benefits not only the pregnant woman but also the fetus and the newborn; thus, vaccines are beneficial in pregnancy for (i) preventing maternal morbidity and mortality; (ii) reducing the risk of fetal infection; and (iii) providing passive immunity to the newborn. Therefore, it is necessary to redouble efforts in public health involvement and education to optimize vaccination and value its benefits for maternal and perinatal health (2).

This is the least that should be expected during the COVID-19 pandemic period. Physiological cardiorespiratory adaptations mean an increased risk of morbidity and mortality in pregnant women; similarly, coagulation system changes during pregnancy and puerperium represent a greater risk for thromboembolic phenomena (3). Both respiratory and thromboembolic complications are important causes of death in pregnant and postpartum women (3).

According to the COVID-19 Brazilian Obstetric Observatory (OOBr), a system that has collected data on the COVID-19 pandemic in Brazilian pregnant and postpartum women and those who have died since March 1, 2020, at the end of July 2021, 11,990 pregnant and postpartum women had been admitted to hospital units owing to SARS-COV-2, with a lethality rate of 12.7% (4). On the other hand, the SEADE Foundation showed that the lethality rate of this virus in the general population was 2.8%, a number significantly lower than that observed in the obstetric population (5,6). These numbers had already been confirmed in other countries, showing that, considering women with symptomatic disease, the adjusted hazard ratio in pregnant women was 3.0 for the need for intensive care unit admission, 2.9 for the need for mechanical ventilation, and 1.7 for death compared to non-pregnant women of similar age who had the disease (7). Furthermore, pregnant women with severe or critical coronavirus infection are at increased risk of stillbirth and premature birth. Some studies of pregnant women hospitalized with COVID-19 showed that the risk of premature birth (elective or spontaneous) reached 25% of pregnancies, with rates of up to 60% in women with severe disease presentation (8).

Therefore, national governments should urgently consider these numbers when thinking about public policies to fight the pandemic, investing resources, and allocating efforts to protect this specific population group as a matter of priority. Law 14,190/21, approved by the Brazilian National Congress and sanctioned by the President of the Republic, includes pregnant, breastfeeding, and postpartum women in the list of priority groups for vaccination against COVID-19 (9). It is a correct, necessary, and fundamental measure to protect pregnant and postpartum women.

The Sinovac^®^ / Coronavac^®^, AstraZeneca^®^, Pfizer-BioNTech^®^, and Janssen^®^ vaccines have been approved for use in Brazil. Of these, Sinovac^®^ / Coronavac^®^ and Pfizer-BioNTech^®^ are approved for use in pregnant and postpartum women, as they have shown a safety profile for women, fetuses, and newborns and have met the efficacy criteria stipulated by the World Health Organization. Sinovac^®^ / Coronavac^®^ showed an efficacy of 50.4% in preventing the disease and 78% in preventing moderate and severe cases. Pfizer-BioNTech^®^ showed 95% efficacy. The analysis of these data, especially regarding efficacy, adverse events, risk of maternal and fetal morbidity, and mortality associated with COVID-19 evidences the need to vaccinate pregnant women (6).

The next step and a key point for the success of the vaccine campaign is the acceptance of the recommended vaccines by pregnant women, thereby improving maternal immunization rates and, consequently, decreasing the risks presented above. In this sense, certain groups of the pregnant population have lower adherence to the vaccine program. National and international studies (10-14) have shown that adherence to vaccination is less likely in pregnant women with lower socioeconomic status and education and from racial and/or ethnic minorities. This non-adherence observed in some segments of the population may be associated, at least in part, with sociocultural and psychological factors that influence vaccine acceptance (15-16).

Factors that could justify this lower adherence are the belief that the vaccine may pose a personal threat, associated with disbelief in the effectiveness of measures to reduce this threat (17,20). Furthermore, other issues that can influence adherence to the vaccine program are perceived benefits, personal experiences with other vaccines, and confidence in the vaccine’s efficacy.

As expected, there was a significant decrease in the number of cases and deaths in Brazilian pregnant women after this population started being vaccinated. However, two important facts should be considered. First, the reduced number of cases and deaths in pregnant and postpartum women also coincided with decreased cases in the entire Brazilian population. Second, 668,755 pregnant or postpartum women have been vaccinated so far, according to OOBr data updated on August 4, 2021, and considering the first dose and full vaccination, corresponds to less than 30% of the total reported live births in 2019 (2,265,955) in the states with available vaccination data (4).

This demonstrates the importance of measures that include pregnant and postpartum women in the high-risk group and, therefore, as a priority, of the need to guide health teams in the importance of clarifying patients and prescribing the vaccine and of implementing educational programs to encourage these women to get vaccinated. Physicians should recommend COVID-19 vaccination not just for pregnant women, but also for those who are planning to become pregnant or breastfeeding their children. All health care professionals are responsible for being involved in developing strategies to overcome patients’ distrust by providing scientific evidence through robust educational campaigns.

COVID-19 infection has a great potential for morbidity and mortality, especially in pregnant and postpartum women. Vaccination is a strategy of great importance in this context to reduce both maternal and fetal changes, especially in Brazil, where maternal death rates due to COVID-19 are extremely high.

## AUTHOR CONTRIBUTIONS

The authors contributed equally to the study. They were responsible for the conception and design, manuscript writing and revision, and approval of the final version of the manuscript.
